# PhoP: A Missing Piece in the Intricate Puzzle of *Mycobacterium tuberculosis* Virulence

**DOI:** 10.1371/journal.pone.0003496

**Published:** 2008-10-23

**Authors:** Jesús Gonzalo-Asensio, Serge Mostowy, Jose Harders-Westerveen, Kris Huygen, Rogelio Hernández-Pando, Jelle Thole, Marcel Behr, Brigitte Gicquel, Carlos Martín

**Affiliations:** 1 Grupo de Genética de Micobacterias, Departamento de Microbiología, Facultad de Medicina, Universidad de Zaragoza, Zaragoza, Spain; 2 Division of Infectious Diseases and Medical Microbiology, Montreal General Hospital, Montreal, Canada; 3 Central Veterinary Institute, Lelystad, The Netherlands; 4 WIV-Pasteur Institute Brussels, Brussels, Belgium; 5 Experimental Pathology Section, Department of Pathology, National Institute of Medical Sciences and Nutrition “Salvador Zubiràn”, Mexico City, Mexico; 6 TuBerculosis Vaccine Initiative, Lelystad, The Netherlands; 7 Unité de Génétique Mycobactérienne, Institut Pasteur, Paris, France; 8 CIBER Enfermedades Respiratorias, Mallorca, Illes Balears, Spain; Centre for DNA Fingerprinting and Diagnostics, India

## Abstract

Inactivation of the transcriptional regulator PhoP results in *Mycobacterium tuberculosis* attenuation. Preclinical testing has shown that attenuated *M. tuberculosis phoP* mutants hold promise as safe and effective live vaccine candidates. We focused this study to decipher the virulence networks regulated by PhoP. A combined transcriptomic and proteomic analysis revealed that PhoP controls a variety of functions including: hypoxia response through DosR crosstalking, respiratory metabolism, secretion of the major T-cell antigen ESAT-6, stress response, synthesis of pathogenic lipids and the *M. tuberculosis* persistence through transcriptional regulation of the enzyme isocitrate lyase. We also demonstrate that the *M. tuberculosis phoP* mutant SO2 exhibits an antigenic capacity similar to that of the BCG vaccine. Finally, we provide evidence that the SO2 mutant persists better in mouse organs than BCG. Altogether, these findings indicate that PhoP orchestrates a variety of functions implicated in *M. tuberculosis* virulence and persistence, making *phoP* mutants promising vaccine candidates.

## Introduction

The lifecycle of intracellular pathogens requires adaptation to the environment prevailing in the host tissues either to interact with cells or to survive within them. This is particularly important for *M. tuberculosis* which is transmitted by aerosol route with the lung being the primary organ affected. Once *M. tuberculosis* reaches the alveoli it is engulfed by professional phagocytes such as macrophages. Initially, *M. tuberculosis* is able to replicate within macrophages until a cell-mediated immunity is mounted by the host. Then, macrophages are activated by interferon-γ (IFN-γ) and, are able to control the intracellular growth of *M. tuberculosis* by triggering a hostile environment that includes acidification of the phagosome, lysosome maturation and production of NO and reactive oxygen/nitrogen intermediates. However, the tubercle bacillus has evolved strategies to cope with the macrophage defences which include prevention of the phagosome acidification and the arrest of the phagosome maturation [1]. Surviving bacteria are believed to enter a state of persistence [2] which can be lifelong. This persistent lifestyle is probably a key reason for the success of *M. tuberculosis* as intracellular pathogen. Indeed, one-third of the human population is latently infected with the bacilli, which represent an important niche.

The ability to persist for long periods in the host depends largely on the capacity of *M. tuberculosis* to acquire and utilize nutrients from the macrophage phagosome. *M. tuberculosis* switches metabolic pathways to utilise fatty acids rather than carbohydrates during persistent infection [3], [4]. In addition, *M. tuberculosis* likely encounters a hypoxic environment during latent infection. The tubercle bacillus is able to elicit an initial hypoxic response through the transcriptional regulation of the dormancy regulon [5], [6]. Following the initial adaptation to oxygen deprivation, long-term survival of *M. tuberculosis* is accomplished by an enduring hypoxic response (EHR) which consists of a transcriptional response much larger than the dormancy regulon and maintained for a much longer period [7]. On the other hand, bacterial exposure to the harsh phagosomal ambience requires a stress response to deal with the oxidative, nitrosative and acidic stresses found in macrophages. Overall, in order to successfully survive intracellularly, *M. tuberculosis* possesses regulatory networks to adapt its metabolism to the environment prevailing within phagosomes. Some works have studied the bacterial transcriptome to reveal the intracellular response of *M. tuberculosis* [4], [8]–[10].

In this work we have focused on the *phoP* gene, which encodes the transcriptional regulator of the two-component system (2CS) PhoPR. Inactivation of *phoP* results in high attenuation of *M. tuberculosis*. The mutant is impaired to grow in macrophages and BALB/c mice; however, it is not completely eliminated and persists in *in vitro* cultured-macrophages and also in mouse organs [11]. This attenuated phenotype and the ability to persist in the host probably contribute to confer a protective immunity in mice and guinea pigs that results in a higher level of protection against tuberculosis than that conferred by the current BCG vaccine strain [12]. Further supporting the role of PhoP in virulence regulation, very recent works have demonstrated that a point mutation in PhoP contributes to avirulence of the H37Ra strain, since this mutation abrogates secretion of the ESAT-6 antigen and the synthesis of acyltrehalose-based lipids in this strain [13]–[15].

In this work we compare both the transcriptome and the proteome of *M. tuberculosis* wild type with a *phoP* mutant to characterize the PhoP regulon, and we test the antigenic capacity and persistence of the *phoP* mutant in mice model. Our results strongly suggest that PhoP controls essential processes for virulence and persistence in *M. tuberculosis*.

## Results

### Identification of the PhoP regulon by transcriptome and proteome approaches

**Figure 1:**
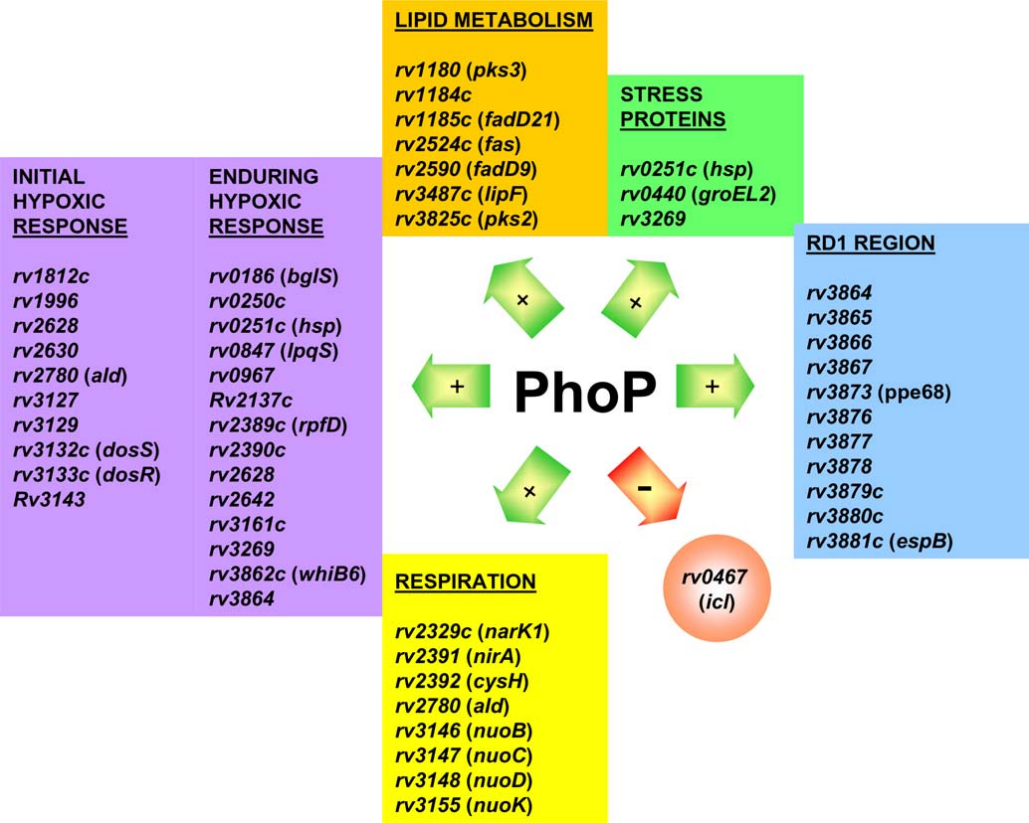
The *M. tuberculosis* PhoP regulon. The PhoP regulon was identified by comparing transcriptional profiles of the *M. tuberculosis* wild type and the *phoP* mutant using DNA microarrays. Some of the more relevant genes to virulence and intracellular survival are listed and grouped by function. Green and red arrows indicate genes whose expression is positively or negatively regulated by PhoP, respectively.

**Figure 2:**
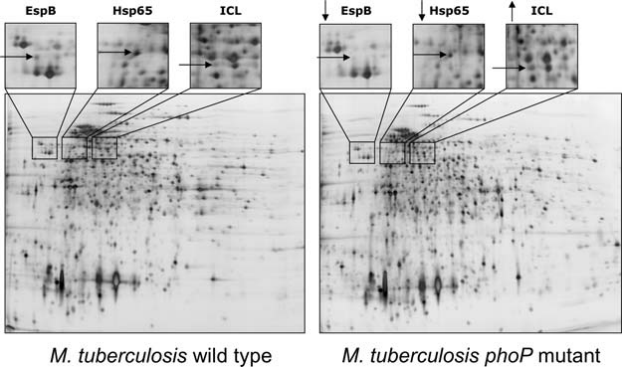
Protein expression patterns of *M. tuberculosis* and the *phoP* mutant. Areas of 2D-polyacrylamide gels show differences in the protein expression patterns between the wild type strain and the *phoP* mutant. Spots that showed at least three-fold differential expression across triplicate gels were selected for identification by mass spectrometry. EspB and Hsp65 are more expressed in the wild type strain while ICL shows a higher expression in the *phoP* mutant. The vertical arrows indicate decreased (↓) or increased (↑) expression in the *M. tuberculosis phoP* mutant relative to the parent strain.

In a global approach to characterize the PhoP regulon we compared the transcriptome of an *M. tuberculosis* clinical isolate with its *phoP* mutant [16]. Seventy-eight genes - approximately 2% of the coding capacity of the *M. tuberculosis* genome - showed significant differences between both strains (Table S1). In our transcriptomic analysis, the *phoP* gene itself appears downregulated in the mutant; this serves as an excellent internal control and provides confidence in the results. Additionally, down-regulation of the adjacent *phoR* gene strongly supports our previous observations that both genes are transcribed in an operon [17]. Genes positively regulated by PhoP include those required for hypoxia adaptation, genes involved in aerobic/anaerobic respiration, genes within the Region of Difference 1 (RD1), genes encoding stress proteins and genes involved in lipid metabolism. Amongst the few genes negatively regulated by PhoP, we found the *icl*-*fadB2*-*umaA1* operon (Figure 1). In a complementary approach to identify genes regulated by PhoP we compared the protein expression patterns of the wild type strain and the *phoP* mutant. Analysis from two sets of 2D electrophoresis gels revealed that ICL, EspB - an antigenic protein encoded in the extended RD1 (extRD1) region - and stress proteins such as Hsp65 (GroEL2) and alpha crystallin (HspX or Acr) are differentially expressed between both strains. In agreement with the microarray data, alpha crystallin, EspB, and Hsp65 gave higher expression in the wild type whereas ICL gave higher expression in the *phoP* mutant (Figure 2 and Table S2). Remarkably, a number of PhoP regulated genes have been previously shown to be differentially expressed upon *M. tuberculosis* infection of macrophages and dendritic cells (Table S1). These findings point at PhoP as a regulator of key functions for intracellular survival in *M. tuberculosis*.

### PhoP mediates early and enduring hypoxic responses in *M. tuberculosis*

**Figure 3:**
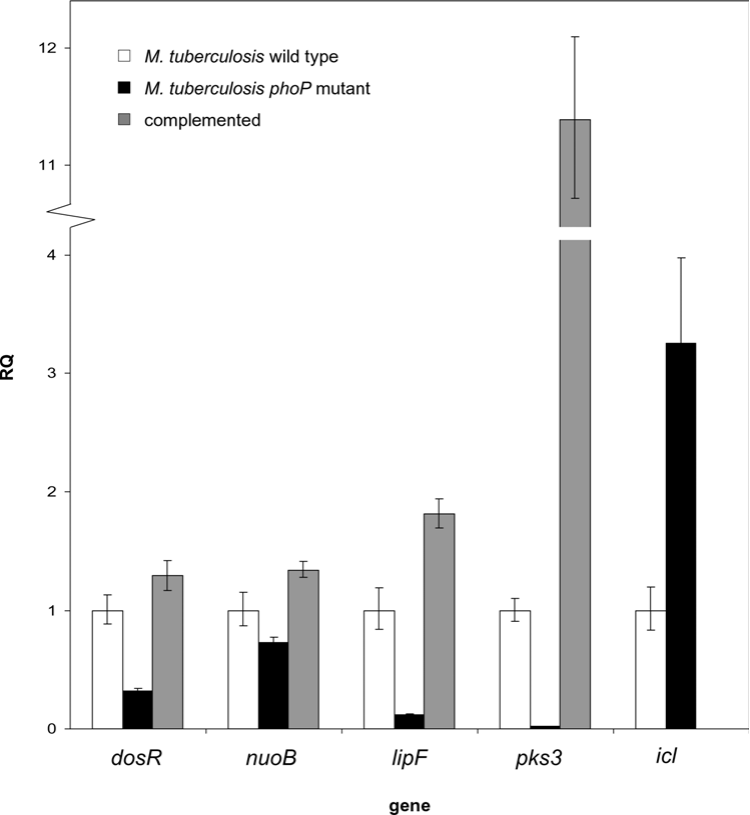
Quantification of gene expression by qRT-PCR. Relative expression levels of the *dosR*, *nuoB*, *lipF*, *pks3* and *icl* genes. The relative quantity (RQ) for each gene in the *phoP* mutant and the complemented strain were calculated with respect to the gene expression levels in the wild type strain. The expression levels of each gene in each strain were normalized to the levels of *sigA* mRNA. Primers and probe sequences for the aforementioned genes as well as for the endogenous control *sigA* are listed in Table S3.

Under the initial hypoxic conditions within macrophages, *M. tuberculosis* enters a dormant state characterized by the induction of the so called dormancy regulon which includes approximately 50 genes [5], 18 under the control of 2CS DosRST [19]–[21]. In this work we show that part of the DosR regulon, including the *dosRS* genes themselves, is under the control of PhoP as indicated as initial hypoxic response in Figure 1. In addition, alpha crystallin - a latency antigen which also belongs to the DosR regulon [5] - also appears downregulated in the *phoP* mutant in our proteome comparison (Table S2). Altogether, these observations indicate that PhoP might regulate the dormancy regulon through crosstalking with DosR. To really confirm that *dosR* is under the control of PhoP, we carried out qRT-PCR analyses. Our results demonstrate that *dosR* expression is reduced in the *phoP* mutant with respect to the wild type strain and, complementation of the *phoP* mutant with the *phoPR* operon restores *dosR* expression to wild type levels (Figure 3). The DosRS 2CS was initially discovered for being higher expressed in the virulent *M. tuberculosis* H37Rv than in its avirulent counterpart H37Ra [22], [23]. Hence, DosRS was initially named DevRS, an acronym for differentially expressed in virulent strain. Here, we demonstrate by qRT-PCR that *dosR* is downregulated in H37Ra with respect to H37Rv (Figure S1). This is probably a consequence of a Ser219Leu mutation in PhoP from H37Ra. On the other hand, it has been recently demonstrated that following the initial adaptation to hypoxia through the DosR regulon, *M. tuberculosis* initiates an EHR [7]. Interestingly, we show for the first time that PhoP also regulates a subset of genes from the EHR as indicated as enduring hypoxic response in Figure 1. In sum, these findings suggest that PhoP serves as a link between the early and enduring hypoxia responses in *M. tuberculosis*.

### PhoP regulates respiratory functions in *M. tuberculosis*

In order to adapt to fluctuations in the oxygen levels during infection, *M. tuberculosis* switches from aerobic to anaerobic respiration [10], [24]. Here we show for the first time that PhoP positively regulates *nuo* genes from the NADH dehydrogenase operon as described as respiration in Figure 1. This enzymatic complex functions as the primary electron acceptor via oxidation of NADH to NAD^+^. We confirmed by qRT-PCR that the *nuoB* gene is transcribed at lower levels in the *phoP* mutant than in the wild type and the complemented strains (Figure 3). Downregulation of *nuo* genes in the *phoP* mutant indicates that PhoP probably controls the expression of the entire *nuo* operon. NuoG inhibits apoptosis in macrophages and increases virulence in immunocompromised mice [25]. Thus, downregulation of the *nuoG* gene in the *phoP* mutant (Z-Score = 1.88) would contribute to both, attenuation and increased apoptosis, which in turn would result in better antigen presentation [26]. We also show for the first time that PhoP regulates the expression of the enzyme alanine dehydrogenase (*ald*). This enzyme contributes to maintain the NADH pool by recycling NAD^+^ through the conversion of pyruvate to alanine when oxygen, as a terminal electron acceptor, becomes limiting [27]. Additionally, PhoP controls the genes involved in utilisation of nitrogen and sulphur sources in oxygen limiting conditions such as the nitrite transporter *narK1* and the sulphur reduction operon *nirA-cysH* [28].

### PhoP regulates genes within the RD1 region required for virulence and ESAT-6 secretion

**Figure 4:**
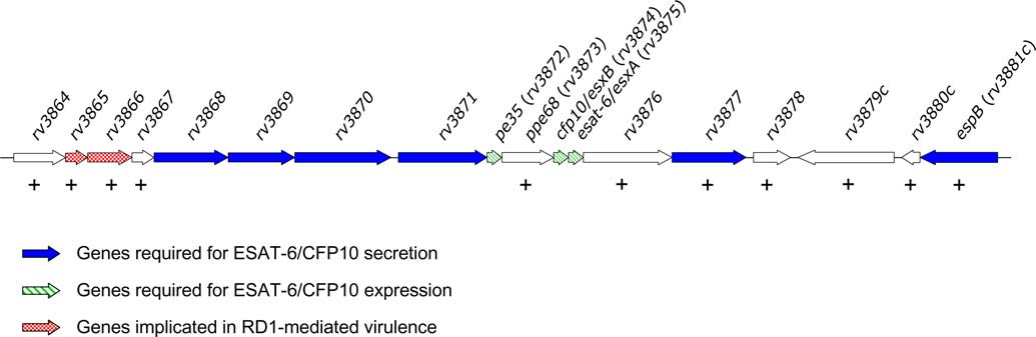
Schematic representation of the PhoP-regulated genes within the extRD1 region. The extRD1 region includes genes essential for ESAT-6/CFP10 secretion (blue), genes essential for ESAT-6/CFP10 expression (green) and genes implicated in RD1-mediated virulence (red). Genes identified as positively regulated by PhoP are indicated (+).

RD1 is a genomic region essential for *M. tuberculosis* virulence [29] which is present in virulent members of the *M. tuberculosis* complex but deleted from all BCG vaccines [30]. RD1 encodes the dedicated secretion system ESX-1, which assures export of the major T-cell antigen complex ESAT-6/CFP10 [31]–[33]. Here, we show, as a novel finding, that PhoP positively regulates many genes within the RD1 as described in Figure 1. Some of these genes are required for RD1-mediated virulence (Figure 4) [31] and their downregulation in the *M. tuberculosis phoP* mutant probably contributes to attenuation. Our previous studies with the *M. tuberculosis phoP* mutant SO2 have demonstrated the presence of ESAT-6 in cell-free extracts [12] but not in the culture filtrate [14]. From our data of the PhoP regulon in *M. tuberculosis*, we attempted to infer the mechanism by which PhoP influences ESAT-6 secretion. Recent works have demonstrated the requirement of EspB for ESAT-6/CFP10 co-secretion [34], [35]. The *espB* gene lies within the extRD1 region and also belongs to the PhoP regulon identified in this work, since it appears downregulated in the *M. tuberculosis phoP* mutant in our proteome and transcriptome comparisons (Figure 1 and Figure 2). Thus, decreased expression of EspB in the *phoP* mutant might be responsible for the lack of ESAT-6 export in this strain [14]. Additionally, it has been recently identified a novel transcription factor encoded by the *rv3849* gene which promotes secretion of ESX-1 substrates, including ESAT-6 [36], and hence was renamed EspR, an acronym for ESX-1 secreted protein regulator. Remarkably, the *rv3849* gene appears downregulated in the *M. tuberculosis phoP* mutant in our transcriptome comparison (Table S1). Taken together, the co-ordinate regulation of EspB and EspR by PhoP would contribute to unravel a novel transcriptional mechanism for the control of ESAT-6 secretion.

### PhoP regulates the stress response in *M. tuberculosis*

Stress proteins play an important role for intracellular survival protecting *M. tuberculosis* against oxidative, nitrosative and/or acidic stresses [4]. Our transcriptome comparison shows that three genes encoding stress proteins are positively regulated by PhoP as indicated in Figure 1. Moreover, our proteomics studies indicate that, in addition to alpha crystallin, the stress protein Hsp65 appears downregulated in the *phoP* mutant (Figure 2). The global control of stress proteins throughout the genome suggests that PhoP co-ordinately regulates the expression of stress-inducible genes.

### PhoP controls genes of the lipid metabolism downregulated in the avirulent strain H37Ra

Previous works have demonstrated that inactivation of PhoP abrogates the synthesis of acyltrehalose-based lipids [16], [37]. Our transcriptome comparison shows that PhoP positively regulates genes implicated in the lipid metabolism as indicated in Figure 1. Additionally to *pks3*, *rv1184c*, *fadD21* and *pks2* which participate in the synthesis of acyltrehalose-based lipids [16], we found that PhoP regulates expression of *lipF* coding for a lipid esterase required for virulence in mice [38] and *fadD9* which encodes a hypothetical fatty acid-CoA ligase. In addition, we found that PhoP controls the *fas* gene encoding a fatty acid synthase which, together with the FAS II system, generates precursors for the synthesis of mycolic acids. Recent work has established a role for PhoP in *M. tuberculosis* H37Ra attenuation, since the Ser219Leu mutation in PhoP is responsible for the lack of acyltrehalose-based lipids in this strain [13]. Indeed, some genes of the PhoP regulon are downregulated in H37Ra with respect to H37Rv [39]. We confirmed by qRT-PCR that *lipF* and *pks3* expression is reduced in both, H37Ra (Figure S1) and the *M. tuberculosis phoP* mutant (Figure 3). Complementation of the mutant with the *phoP* gene restored expression of these genes to wild type levels (Figure 3).

### Expression of the persistence factor ICL is increased in the *M. tuberculosis phoP* mutant

**Figure 5:**
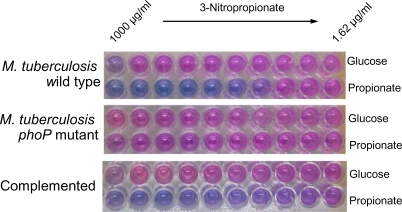
Determination of sensitivity to the ICL inhibitor 3-NP. The wild type, *phoP* mutant and complemented strains were tested for their ability to grow in 7H9 medium supplemented with glucose or propionate as sole carbon sources in the presence of 3-NP. A change from blue to pink coloration is indicative of resazurin reduction and consequently it correlates with bacterial viability. No differences in 3-NP sensitivity were observed when the wild type, the *phoP* mutant or the complemented strains were grown in glucose as unique carbon source. The *phoP* mutant is less sensitive to 3-NP than the parental strain when grown in propionate as sole carbon supplier, indicating higher levels of ICL expression in the mutant. Complementation of the mutant with the *phoPR* operon renders bacteria as susceptible to 3-NP as the parental strain when propionate is the unique carbon source.

In addition to hypoxia adaptation, the ability of *M. tuberculosis* to persist in the host depends largely on the capacity to utilise fatty acids during infection [3], [4]. Fatty acids are degraded to acetyl-CoA and propionyl-CoA subunits. The glyoxylate pathway is required for carbon anaplerosis of acetyl-CoA during starvation, while the methylcitrate cycle is required for propionyl-CoA metabolism and detoxification [40], [41]. Both pathways share the enzyme ICL [40]. Due to its role in the utilisation of intraphagosomal carbon sources, ICL has been shown to be required for persistence and virulence of *M. tuberculosis* in macrophage and mice [42]. Our results from the transcriptome and the proteome comparison consistently show as a novel finding that PhoP negatively regulates the expression of ICL as shown in Figure 1 and Figure 2. qRT-PCR studies showed that *icl* expression was higher in the *phoP* mutant than in the wild type strain (Figure 3). Unexpectedly, complementation did not restore expression of the *icl* gene to wild type levels. Consequently *in vitro* studies were performed to check the complementation of ICL expression. We tested the ability of the ICL inhibitor 3-nitropropionate (3-NP) [43] to block growth of the wild type and the *phoP* mutant in media containing either glucose or propionate as the sole carbon sources. No differences in 3-NP sensitivity were observed when the wild type or the *phoP* mutant strains were grown in glucose as carbon substrate. However, when bacteria were forced to induce *icl* expression to metabolise propionate as unique carbon source [40], the *phoP* mutant was less sensitive to 3-NP when compared to wild-type bacteria (Figure 5). In addition, complementation of the mutant with the *phoPR* operon restored 3-NP sensitivity (Figure 5). These results demonstrate that the *phoP* mutant is better pre-adapted than the parental strain to survive under environmental conditions which require *icl* expression, and this may possibly due to the higher ICL levels in this mutant.

### Immunological properties of the *phoP* mutant

**Figure 6:**
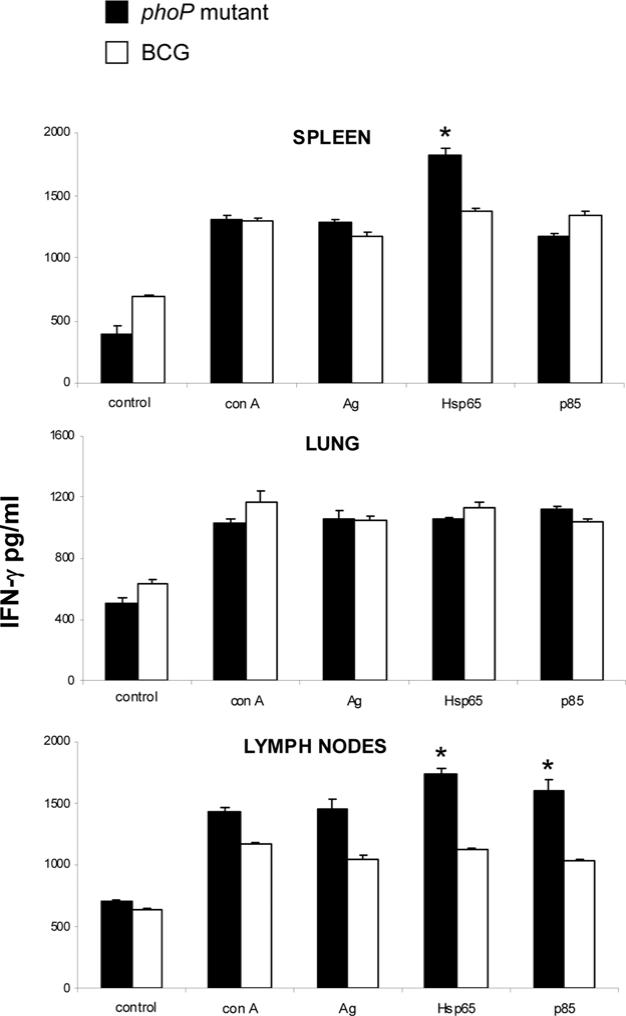
Hsp65- and Ag85A-specific responses exhibited by mice immunised with *M. tuberculosis phoP* mutant and BCG. Cells from spleen, lungs and lymph nodes from mice immunised with either BCG or the *phoP* mutant were stimulated with Hsp65 or Ag85A (p 85) and IFNγ production was measured by ELISA. Bars represent mean and SD from two separate experiments. Asterisks indicate significant differences in IFN-γ production. A higher percentage of Hsp65-specific cells is found in spleen and lymph nodes from mice immunized with SO2 when compared with BCG-immunised mice. Lymph nodes from SO2-immunised mice contained a higher fraction of cell responding to Ag85 in comparison with BCG-immunised mice.

**Figure 7:**
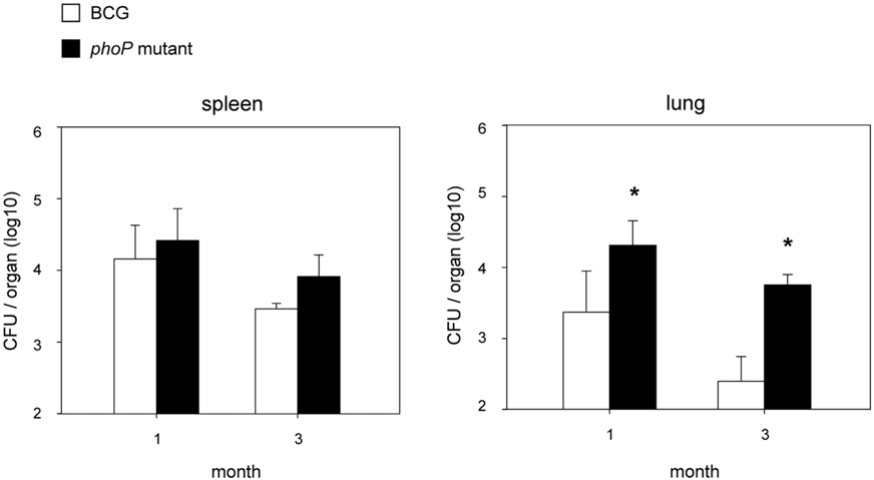
Persistence of BCG and the *M. tuberculosis phoP* mutant in BALB/c mice. Animals were intravenously infected with either BCG or the SO2 *phoP* mutant. Bars represent mean and SD of log_10_ CFUs recovered from spleen and lungs of inoculated animal at 1 and 3 months after the initial infection. Asterisks indicate significant differences in CFU counts. We were unable to compare persistence of the SO2 *phoP* mutant with the wild type since the latter strain kills animals by day 60 post infection (data not shown).

In preclinical studies we have previously shown that the SO2 *phoP* mutant is more attenuated than BCG and confers protective immunity against tuberculosis in mice and guinea pigs [12]. In order to test whether SO2 was able to elicit antigen-specific responses comparable to the BCG vaccine, BALB/c mice were immunized with both strains and one month after the initial inoculation, Ag85A- and Hsp65-specific responses were measured by ELISA. We observed that even if both strains present similar antigenic capacity, mice immunised with the SO2 *phoP* mutant exhibit better anti-Hsp65 and anti-Ag85A responses than BCG-vaccinated mice (Figure 6). Additionally, given that a number of vaccine candidates in clinical and preclinical studies are based on Ag85-complex [44], immunity to this antigen is a substantial benefit for the SO2 vaccine candidate. Alternatively, given that persistence in the host could be a potential advantage for a live attenuated vaccine, together with the evidence that ICL is required for chronic persistence of *M. tuberculosis* in mice [42] led us to study the persistence of the SO2 *phoP* mutant. BALB/c mice were intravenously inoculated with either BCG or SO2. Both BCG and the *phoP* mutant could be readily detected in spleen and lungs at 1 month after immunization, but BCG was more rapidly cleared from spleen and particularly from lungs than SO2 after 3 months (Figure 7). The increased persistence exhibited by the SO2 *phoP* mutant could result in prolonged exposure to the immune system and consequently in long-term immunogenicity.

## Discussion

In this work we identify the PhoP regulon in a clinical isolate of *M. tuberculosis*. Although the PhoP regulon has been previously studied in H37Rv demonstrating regulation of genes involved in complex lipid biosynthesis [37], in this work we extend the PhoP regulatory network with genes essential for virulence and persistence in *M. tuberculosis* not previously described. Our results demonstrate that PhoP positively regulates six major circuits: i) the early and enduring hypoxic responses, ii) functions for aerobic and anaerobic respiration, iii) genes within RD1 required for virulence and ESAT-6 secretion, iv) the stress response, v) the lipid metabolism and vi) the *M. tuberculosis* persistence through the control of ICL (Figure 1).

Insights into the transcriptional response to the macrophage environment have served to identify functions required for the intracellular lifestyle of *M. tuberculosis* [4], [8]–[10]. Remarkably, the six transcriptional networks under the control of PhoP identified in this work appears differentially expressed in response to macrophage infection (Table S1), indicating that PhoP might control key functions for intracellular survival.

### Hypoxia response

We demonstrate that PhoP mediates the early hypoxic response in *M. tuberculosis* through crosstalk with DosR. Accordingly, part of the DosR regulon is downregulated in the *phoP* mutant (Figure 1). It has been demonstrated that the DosR regulon is induced in response to macrophage infection [4], [8]–[10], supporting the role of these genes in the adaptation to oxygen deprivation and exposure to oxidative radicals through sensing NO, CO or low O_2_ found within macrophages [20], [21], [45]. Some genes of the DosR regulon are known to be important T-cell antigens [46] and recent work has demonstrated that the attenuated BCG vaccine is defective for induction of two dormancy genes, *narK2* and *narX* [47]. Conversely, the Beijing strain associated with epidemic spread and enhanced virulence has been shown to constitutively express the DosR regulon [48], which suggests a possible role for the DosR regulon in virulence and consequently, downregulation of DosR in the *phoP* mutant could contribute to the attenuated phenotype of this strain.

### Aerobic and anaerobic respiration

PhoP regulates the synthesis of some components of the NADH dehydrogenase complex (Figure 1), the primary electron acceptor of the aerobic respiratory chain. Downregulation of *nuo* genes has been reported in response to macrophage infection [4], [9], [10], indicating the shift in the respiratory state from aerobic to micro-aerobic or anaerobic. PhoP also controls the expression of the *ald* gene (Figure 1). Ald might be involved in NADH regeneration under limiting O_2_ environments [27] and hence, it appears induced in *M. tuberculosis* upon infection of macrophage and dendritic cells [4], [10]. The *ald* gene is upregulated in *M. tuberculosis* upon nutrient starvation [49] and in *Mycobacterium marinum* during persistent infection [50], which suggest a role for this enzyme in hypoxia-mediated persistence [51]. In addition, it has been shown that all BCG strains lack a functional Ald enzyme and this may results in restricted ability of BCG to persist within the host [52]. Genes of the anaerobic respiration belonging to the PhoP regulon include the nitrite transporter *narK1* and the sulphur reduction operon, *nirA*-*cysH* (Figure 1), all of which have been differentially expressed in response to macrophage infection [8], [9], [53] and further supporting the metabolic shift from aerobic to anaerobic respiration. CysH could have a secondary role in protecting *M. tuberculosis* during the chronic phase of infection [54]. Indeed, an *M. tuberculosis cysH* mutant is attenuated and generates protective efficacy against tuberculosis infection equivalent to that of BCG [55]. In this context, downregulation of the *cysH* gene in the *phoP* mutant could contribute to the attenuated phenotype and the protection against disease displayed by this strain.

### RD1-mediated virulence

A number of works supports the role of the RD1 region in virulence: complementation of BCG with RD1 increases virulence in mice [29] and conversely, deletion of RD1 in *M. tuberculosis* produces attenuation [56], [57]. Concretely, the *rv3865* and *rv3866* genes are involved in RD1-mediated virulence [31]. Thus, downregulation of these genes in the *phoP* mutant could contribute to attenuation. On the other hand, the *espB* homolog in the fish pathogen *M. marinum* is required for virulence and intracellular growth in infected macrophages [35], [58]. Further supporting the role of EspB in virulence, it has been shown that this protein is absent from the attenuated BCG vaccine [59].

### Stress response

Molecular chaperons play a possible role in protecting *M. tuberculosis* against the oxidative radicals produced in phagosomes and thus, their expression is upregulated within macrophages [4], [8], [60]. The PhoP regulon includes genes of the stress response (Figure 1). In addition, the *phoP* gene itself appears upregulated under heat-stress [61]. We also demonstrate that even though the *phoP* mutant displays decreased expression of stress proteins, this strain is able to elicit an anti-Hsp65 response comparable to that of the vaccine strain BCG (Figure 6). This might result as a consequence of the persistent phenotype of the *phoP* mutant, since prolonged exposure of the mutant to the immune system likely results in better anti-Hsp65 responses.

### Lipid metabolism

Some genes of the PhoP regulon required for the synthesis of acyltrehalose-based lipids are upregulated in response to macrophage infection [4], [8]–[10], [62]. This suggests that these lipids might play a role in either virulence or immunomodulatory processes. Other genes from the PhoP regulon which are also upregulated in response to macrophage infection include *lipF* and *fadD9* [4], [8]–[10]. Altogether, these findings suggest that PhoP might control the cell envelope remodelling in response to the intracellular environment.

### Persistence

The ability of *M. tuberculosis* to persist for long period in the infected host is probably the result of a number of metabolic adaptations. Among them, one of the most studied is the anaplerotic utilisation of intracellular carbon sources through the glyoxylate shunt enzyme ICL [40], [42], [63]. Various works coincide to show upregulation of the *icl* gene after *M. tuberculosis* infection of macrophages and dendritic cells [4], [8]–[10], [53], [62], which reflects the key implications for this enzyme in intracellular persistence. In this work, we demonstrate that PhoP negatively regulates *icl* expression by transcriptomic and proteomic comparisons (Figure 1 and Figure 2), as well as in qRT-PCR analyses (Figure 3) and biochemical studies (Figure 5). The increased expression of ICL in the *phoP* mutant could account for the persistent phenotype displayed by this strain upon infection of BALB/c mice (Figure 7).

### General conclussion

Overall, we can conclude that PhoP regulates key functions required for the intracellular survival and persistence of *M. tuberculosis*. Therefore, inactivation of *phoP* results in downregulation of genes required to successfully survive within macrophages and consequently in *M. tuberculosis* attenuation. On the other hand, we provide evidence that ICL is expressed at higher levels in the *phoP* mutant than in the parental strain. Thus, *M. tuberculosis phoP* mutants would be better pre-adapted to persist in the host. Taken together, these observations provide a plausible explanation for the attenuated but persistent phenotype displayed by *phoP* mutants and allow understanding the potential applications as vaccine candidates [64].

## Materials and Methods

### Bacterial strains used in this study

We used the *phoP* deletion mutant previously constructed in the *M. tuberculosis* clinical isolate MT103. This mutant was constructed by replacing an *EcoR*V-*Bcl*I restriction fragment internal to the *phoP* gene with a hygromycin resistance marker [16]. The mutant was complemented with the entire *phoPR* operon using the replicative plasmid pJUZ1K [16]. The *phoP* mutant SO2 constructed in the MT103 strain [11] was used to test the immunological properties. The strains H37Rv [65] and H37Ra (ATCC n° 25177) were also used in this study.

### RNA isolation

The *M. tuberculosis* wild type, the *phoP* mutant and complemented strains were grown until the desired OD_600_ in 7H9-ADC 0.05% Tween 80 at 37°C under aerobic conditions. The RNA from bacterial pellet was stabilised using the RNAprotect Bacteria Reagent (QIAGEN) following manufacturer's recommendations. Cells were resuspended in 1 ml acid phenol∶chloroform (5∶1) and 0.4 ml lysis buffer (0.5% SDS, 20 mM NaAc, 0.1 mM EDTA) and transferred to 2 ml Lysing Matrix B screw-cap tubes containing 0.1 mm silica spheres (Q-BIOgene). Cells were disrupted by three 30 s pulses in a FastPrep homogenizer (Q-BIOgene). After centrifugation, RNA from the supernatant was further extracted with 0.9 ml chloroform∶isoamyl alcohol (24∶1). Total RNA was precipitated with NaAc/isopropanol and washed with 70% ethanol. The extracted RNA was treated with RNase-free DNase (Ambion) and the RNA was then further purified, using the RNeasy kit (Qiagen). DNA contamination was ruled out by lack of amplification products after 35 cycles of PCR and the integrity of the RNA was checked by gel electrophoresis in a 1% agarose gel.

### DNA microarray analysis

Two independent cultures of each, the wild type strain and the *phoP* mutant were grown until OD_600_≈0.45. At this point RNA was prepared and Cy3/Cy5 labeled for use in genome-wide transcription profiling experiments using glass slide microarrays. A Virtek Chipwriter (model SDDC2) was used to print oligonucleotides on Sigmascreen microarray slides (Sigma). Lyophilized 70-mers from the TB Array-Ready Oligo Set (Operon) were resuspended and printed in duplicate as twenty-four 24×24 grids. Duplicate hybridizations were performed for each dye combination (Cy5 vs Cy3 and Cy3 vs Cy5), amounting to 8 independent hybridizations using 4 different biological RNA samples. All spots flagged as misrepresentative (array artefacts, etc.) were analytically ignored. Total spot intensity minus the surrounding background produced a corrected spot intensity. Negative corrected spot intensities were set to +1. Intensity ratios (Cy3/Cy5 or Cy5/Cy3) were determined using corrected spot intensities and log_10_ transformed. Values for each gene were obtained for each array in duplicate (inherent to array design) and averaged. For each array, a representative Z-score, indicative of how many standard deviations a data point lies above or under the population mean, was calculated for each gene. Z-scores for each gene were averaged across the replicates within each experiment to minimize the probability of observing such variations by chance alone. Only genes with average Z-scores ≥2 or ≤−2 were considered as statistically significant.

### quantitative Real-Time PCR (qRT-PCR)

Expand Reverse Transcriptase (Roche) was used to prepare randomly primed cDNA libraries from 1 µg of each RNA sample. cDNA prepared in this manner was diluted 1∶10 prior to use in subsequent qRT-PCR experiments. The primers and FAM-labelled TaqMan probes used in qRT-PCR experiments were designed using the Primer Express Software (Applied Biosystems) and are listed in Table S3. qRT-PCR was carried out in a StepOne Plus instrument (Applied Biosystems) using standard reaction conditions recommended by the manufacturer.

### 2D-electrophoresis and mass spectrometry

For preparation of cellular proteins, 100 ml cultures of the wild type and the *phoP* mutant were grown in 7H9-ADC-0.05% Tween 80 to OD_600_≈0.8 and cells were pelleted by centrifugation. Pellets were washed twice with PBS and then resuspended in cold PBS. To avoid proteolytic degradation protease inhibitors (2.5 µg/ml pepstatin A, 5 µg/ml leupeptin, 25 µg/ml pefabloc and 1 µg/ml aprotinine) were added prior to cell lysis. Mycobacteria were disrupted by sonication using a bioruptor (Diagenode) for ten cycles (45 sec at high power) allowing to cool in an ice-water bath for 1 min between pulses. The proteins were treated with 9 M urea, 70 mM DTT and 2% Triton X-100 to obtain completely denatured and reduced proteins. The mixture was incubated 30 min at room temperature with regular mixing and then centrifuged. The supernatant containing whole-cell protein extracts was filtered through a 0.22 µm-pore-size low protein binding filter. The cellular proteins were separated by 2D gel electrophoresis. First dimension separation was carried out by loading 90 µg onto IEF strips pH4-7 (GE healthcare) followed by isoelectric focusing for 64000 Vhr on the IPGphor (Amersham/GEhealthcare). Second dimension separation was carried out using 20×25 cm polyacrylamide gels and the EttanDALTtwelve separation unit (Amersham) according to the manufacturer's manual. Gels were stained with silver nitrate (Silver stain, Amersham), digitised using a AGFA duoscan T2500 and analysed and compared using PDquest 7.4.0 (Bio-rad). For subsequent MS analysis, gels were rerun with 150 µg proteins and stained with a mass spectrometry compatible silver stain (SilverQuest, Invitrogen). Protein spots of interest were excised from gel and were sent to the Free University in Amsterdam for Mass Spectrometry (4800 MALDI TOF/TOF, Applied Biosystems). Identification of proteins was performed using MALDI-MS peptide mass fingerprinting and Mascot Search (http://www.matrixscience.com) with a species-limited search filter, which restricted the search to *M. tuberculosis* complex.

### Resazurin microtiter assay for determination of sensitivity to 3-NP

Sensitivity to 3-NP was determined by the resazurin assay. 3-NP was dissolved in water and 2-fold serial dilutions of the inhibitor were made in 7H9 AS (5 mg/ml albumin, 0.85 mg/ml NaCl in 7H9 medium) in microtiter plates, being the final 3-NP concentration range 1000-1.62 µg/ml. Cultures of wild type, the *phoP* mutant and complemented strains were grown in 7H9 AS supplemented with 0.1% of either glucose or propionate as sole carbon sources. When OD_600_ of the cultures reached a value of 0.3–0.5, 0.1 ml of each bacterial suspension were added to the microtiter plate containing 0.1 ml dilutions of 3-NP. The plates were incubated for 24 h at 37°C. Then, 20 µl of a 0.01% resazurin solution were added per well, colouring them blue. Plates were incubated at 37°C for additional 24 h. After incubation plates were read for color change from blue to pink, indicative of resazurin reduction by viable bacteria.

### Measurement of T-cell responses against *M. tuberculosis* Hsp65 and Ag85A

Animal work was performed with approval from the local Ethical Committee for Experimentation in Animals in Mexico. Two separate experiments were performed, 8 weeks old male BALB/c mice (4 per group) were immunised by subcutaneous inoculation in the base of tail vein with 8000 CFUs of the BCG Phipps vaccine and 2500 CFUs of the SO2 *phoP* mutant. One month after the initial immunisation, mice were euthanased and cell suspensions were prepared from spleen, lungs and lymph nodes. Cells were stimulated with 5 µg/ml of either Hsp65 or Ag85A for 48 h. Then, IFNγ production in the supernatant was measured by ELISA.

### Infection of BALB/c mice and enumeration of CFUs in mouse organs

BALB/c (H-2^d^) mice were bred in the Animal Facilities of the Pasteur Institute of Brussels, from breeding couples originally obtained from Bantin & Kingman (UK). 8–10 weeks old BALB/c mice (4 per group) were intravenously inoculated with 5×10^5^ CFUs of either the BCG vaccine (GL2 strain) or the SO2 *phoP* mutant. Mice were sacrificed humanely at 1 and 3 months after the initial immunisation. Spleen and lung tissue were aseptically removed and processed to enumerate the number of bacteria. Tissues were homogenised in 10 ml of sterile PBS using a Dounce Homogenizer. Viable counts were performed on serial dilutions of the homogenate, plated on 7H11-OADC Middlebrook agar and enumerated after 3 weeks growing at 37°C. Numbers of CFU/organ were converted to log10 CFU values. Results are reported as mean log_10_ CFU+/−SD of four mice tested individually.

## Supporting Information

Table S1 Whole-genome transcriptional profiling comparing the *M. tuberculosis* wild type strain with the *phoP* mutant. The upper part of the table shows the 74 genes with higher expression in the wild-type than in the mutant strain. The lower part of the table shows the 4 genes with higher expression in mutant than in the wild type. The Z-score, indicative of how many standard deviations a data point lies above or below the population mean, is the average for that gene across 4 DNA microarrays using 2 RNA samples each of wild type and the *phoP* mutant (8 hybridizations in total). Genes are shaded depending on their function. Violet indicates genes from the DosR and Enduring Hypoxic Response regulons. Yellow refers to genes of the respiratory metabolism. Orange indicates genes implicated in lipid metabolism. Green denotes genes encoding stress proteins. Blue refers to genes within RD1. The *icl* gene implicated in *M. tuberculosis* persistence is shaded in red. Gray indicates PhoP-regulated genes identified in previous works for being differentially expressed upon *M. tuberculosis* infection of macrophages and dendritic cells [4], [8]–[10].
(0.45 MB PDF)

Table S2 Mass spectrometry analysis of cellular proteins differentially expressed in *M. tuberculosis* wild type and its *phoP* mutant. A protein is positively identified if the confidence interval is >95%, with at least one sequenced peptide displaying a confidence interval of >99%, and if the experimental molecular weight (MW) and isoelectric point (pI) correspond to the theoretical MW and pI. The fold change in expression is the average for each spot of the triplicate gels in two independent experiments using different biological samples.
(0.01 MB PDF)

Table S3 Primers and probes used in qRT-PCR experiments
(0.01 MB PDF)

Figure S1 Relative expression levels of the *dosR*, *lipF* and *pks3* genes in H37Ra with respect to H37Rv. The expression levels of each gene in each strain were normalized to the levels of *sigA* mRNA. Primers and probe sequences for the aforementioned genes as well as for the endogenous control *sigA* are listed in Table S3.
(0.44 MB PDF)
