# Supplementary material for: PhoP: A Missing Piece in the Intricate Puzzle of Mycobacterium tuberculosis Virulence
Source: PLoS One. 2008 Oct 23;3(10):e3496. doi: 10.1371/journal.pone.0003496 (PMC2566814; doi:10.1371/journal.pone.0003496)
Supplement: Table S1 — Whole-genome transcriptional profiling comparing the M. tuberculosis wild type strain with the phoP mutant. The upper part of the table shows the 74 genes with higher expression in the wild-type than in the mutant strain. The lower part of the table shows the 4 genes with higher expression in mutant than in the wild type. The Z-score, indicative of how many standard deviations a data point lies above or below the population mean, is the average for that gene across 4 DNA microarrays using 2 RNA samples each of wild type and the phoP mutant (8 hybridizations in total). Genes are shaded depending on their function. Violet indicates genes from the DosR and Enduring Hypoxic Response regulons. Yellow refers to genes of the respiratory metabolism. Orange indicates genes implicated in lipid metabolism. Green denotes genes encoding stress proteins. Blue refers to genes within RD1. The icl gene implicated in M. tuberculosis persistence is shaded in red. Gray indicates PhoP-regulated genes identified in previous works for being differentially expressed upon M. tuberculosis infection of macrophages and dendritic cells [4], [8]–[10]. (0.45 MB PDF) [file pone.0003496.s001.pdf]

Table S1

gene name	Rv number	Z-Score	Schnappinger *et al*. 2003	Rohde *et al*. 2007	Fontan *et al*. 2008	Tailleux *et al*. 2008
bglS	Rv0186	2,17				
Rv0250c	Rv0250c	2,64	■			
hsp	Rv0251c	2,14	■	■		
groEL2	Rv0440	2,32		■		
mmpS5	Rv0677c	2,46				
phoP	Rv0757	2,96				
phoR	Rv0758	2,43				
phoY2	Rv0821c	2,25				
lpqS	Rv0847	2,65			■	
Rv0967	Rv0967	2,53				
Rv0968	Rv0968	2,19				
pks3	Rv1180	2,08		■		■
Rv1184c	Rv1184c	3,12	■	■	■	
fadD21	Rv1185c	3,32		■	■	
Rv1217c	Rv1217c	2,14				
Rv1218c	Rv1218c	2,21				
Rv1219c	Rv1219c	2,33				
Rv1639c	Rv1639c	3,75				
Rv1687c	Rv1687c	2,64	■			
Rv1812c	Rv1812c	2,17				
Rv1986	Rv1986	2,51	■			
Rv1996	Rv1996	2,53	■			
Rv2137c	Rv2137c	2,61			■	
narK1	Rv2329c	2,82		■	■	
Rv2376c	Rv2376c	2,65		■		
Rv2389c	Rv2389c	2,47			■	
Rv2390c	Rv2390c	3,33		■	■	
nirA	Rv2391	5,14			■	
cysH	Rv2392	3,82			■	
Rv2393	Rv2393	3,14			■	
PE_PGRS	Rv2396	4,08		■		
fas	Rv2524c	2,12				
fadD9	Rv2590	3,14	■	■	■	■
Rv2621c	Rv2621c	2,03	■	■		
Rv2628	Rv2628	2,24	■	■		
Rv2630	Rv2630	2,08	■	■		
Rv2641	Rv2641	2,48				
Rv2642	Rv2642	2,14	■			
35kd_ag	Rv2744c	2,21	■			
ald	Rv2780	2,19	■			■
Rv3127	Rv3127	2,51	■	■		
Rv3129	Rv3129	3,44	■			
dosS	Rv3132c	2,19	■	■		■
dosR	Rv3133c	2,25		■	■	■
PPE	Rv3135	4,08			■	
PPE	Rv3136	2,62		■		
Rv3137	Rv3137	2,74		■		
fadB4	Rv3141	2,11				
Rv3143	Rv3143	2,50				
nuoB	Rv3146	2,13	■		■	■
nuoC	Rv3147	2,03			■	■
nuoD	Rv3148	2,01	■		■	■
nuoK	Rv3155	2,14			■	■
Rv3161c	Rv3161c	2,05				
Rv3197	Rv3197	2,54	■			
Rv3269	Rv3269	2,18				
ctpC	Rv3270	2,36				
PE	Rv3477	3,20		■	■	
lipF	Rv3487c	3,17		■		
Rv3822	Rv3822	2,72			■	
pks2	Rv3825c	2,23		■		■
Rv3849	Rv3849	3,36				
whiB6	Rv3862c	2,59		■		
Rv3864	Rv3864	2,13				
Rv3865	Rv3865	2,11				
Rv3866	Rv3866	2,11			■	
Rv3867	Rv3867	2,18				
PPE	Rv3873	2,37	■		■	
Rv3876	Rv3876	2,46				
Rv3877	Rv3877	2,40				
Rv3878	Rv3878	2,89				
Rv3879c	Rv3879c	2,62				
Rv3880c	Rv3880c	3,33				
espB	Rv3881c	2,41				

			Schnappinger *et al*. 2003	Rohde *et al*. 2007	Fontan *et al*. 2008	Tailleux *et al*. 2008
icl	Rv0467	-2,71	■	■	■	■
fadB2	Rv0468	-2,82	■	■	■	
umaA1	Rv0469	-2,10		■	■	
PE	Rv1040c	-2,70				
