# Supplementary material for: PhoP: A Missing Piece in the Intricate Puzzle of Mycobacterium tuberculosis Virulence
Source: PLoS One. 2008 Oct 23;3(10):e3496. doi: 10.1371/journal.pone.0003496 (PMC2566814; doi:10.1371/journal.pone.0003496)
Supplement: Table S2 — Mass spectrometry analysis of cellular proteins differentially expressed in M. tuberculosis wild type and its phoP mutant. A protein is positively identified if the confidence interval is >95%, with at least one sequenced peptide displaying a confidence interval of >99%, and if the experimental molecular weight (MW) and isoelectric point (pI) correspond to the theoretical MW and pI. The fold change in expression is the average for each spot of the triplicate gels in two independent experiments using different biological samples. (0.01 MB PDF) [file pone.0003496.s002.pdf]

**Table S2**

| Spot | Up or unique | Strain | Fold change (1) | Fold change (2) | theoretical | | experimental | | MS |
|---|---|---|---|---|---|---|---|---|---|
| | | | | | pI | KDa | pI | KDa | |
| 604 | Up | wild type | 146.8 | 11.4 | 4.75 | 47.6 | 4.4 | 45 | Rv3881c (*espB*) |
| 2009 | Up | wild type | 210.2 | 3.6 | 5 | 16.1 | 4.8 | 12 | Rv2031c (*hspX*) |
| 2604 | Up | wild type | 2.4 | 5.2 | 4.85 | 57 | 4.7 | 45 | Rv0440 (*groEL2*) |
| 3611 | Up | *phoP* mutant | 4.8 | 2.4 | 5.03 | 47 | 5 | 43 | Rv0467 (*icl*) |
