# Supplementary material for: PhoP: A Missing Piece in the Intricate Puzzle of Mycobacterium tuberculosis Virulence
Source: PLoS One. 2008 Oct 23;3(10):e3496. doi: 10.1371/journal.pone.0003496 (PMC2566814; doi:10.1371/journal.pone.0003496)
Supplement: Table S3 — Primers and probes used in qRT-PCR experiments (0.01 MB PDF) [file pone.0003496.s003.pdf]

Table S3

**PCR primers**	**Sequence**
sigA	5’-CGCGCCTACCTCAAACAGAT-3’
	5’-CCTCTTCCTCGGCGTTGA-3’
rv3877	5’-CCGTTGGTCGAGGATGTCATC-3’
	5’-CCCCACAAAGCGATTCAATGC-3’
dosR	5’-CGCGCGCCGTCAA-3’
	5’-CGCGGCCCGATTGTC-3’
icl	5’-CTCGCGGCCGATGTG-3’
	5’-CGTCGGTACGGGCGATC-3’
nuoB	5’-CCGGGTCAGCCAGAAGAT-3’
	5’-TTCGGCTCCGCCATCTG-3’
pks3	5’-AGCAGCTGATGGCTTCGT-3’
	5’-ATCCGGTAGCCGCTTCAG-3’
lipF	5’-AAGCAGGACCATCCCAACATC-3’
	5’-CCGCCAAGGCATCGAATG-3’
**TaqMan probes**	**Sequence**
sigA	5’-CAGCGCTACCTTGCC-3’
rv3877	5’-ACGAGTCACCTGAGTTCG-3’
dosR	5’-CCAGCGCCCACATCT-3’
icl	5’-ACCACCGTGGGAACAT-3’
nuoB	5’-CTGCGCCAGATCTAT-3’
pks3	5’-CACGCACCCCTCACCG-3’
lipF	5’-CCGACGCGATGTTTC-3’
