# Supplementary figures and images for: PhoP: A Missing Piece in the Intricate Puzzle of Mycobacterium tuberculosis Virulence
Source: PLoS One. 2008 Oct 23;3(10):e3496. doi: 10.1371/journal.pone.0003496 (PMC2566814; doi:10.1371/journal.pone.0003496)

Figure S1

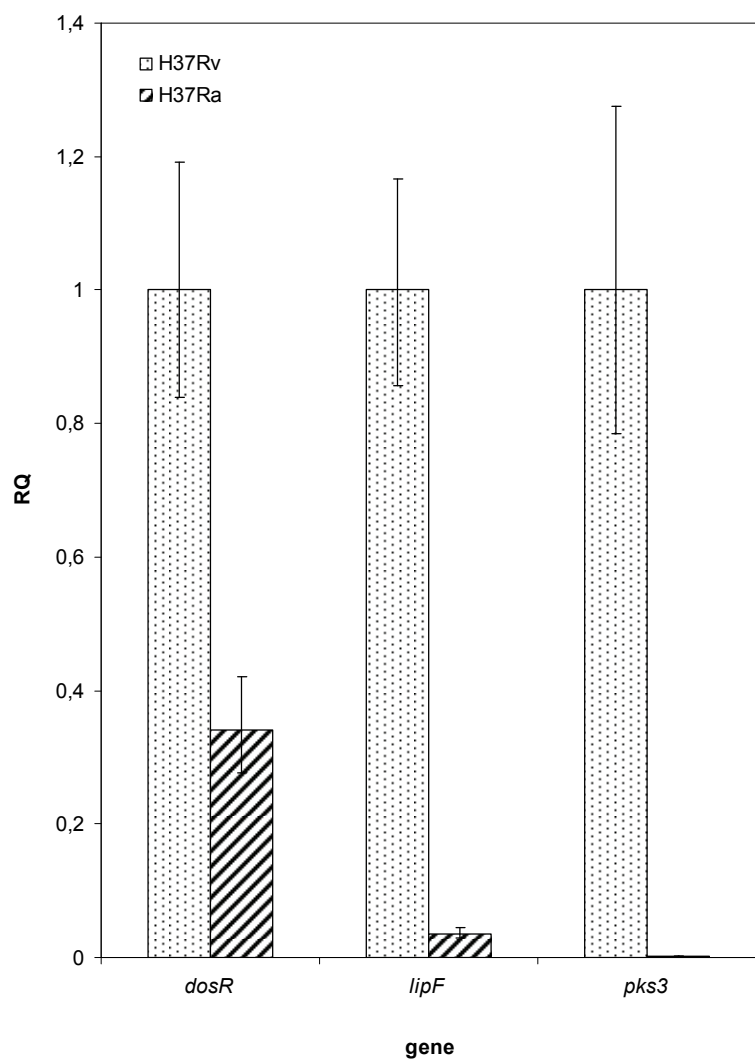

Supplement: Figure S1 — Relative expression levels of the dosR, lipF and pks3 genes in H37Ra with respect to H37Rv. The expression levels of each gene in each strain were normalized to the levels of sigA mRNA. Primers and probe sequences for the aforementioned genes as well as for the endogenous control sigA are listed in Table S3. (0.44 MB PDF) [file pone.0003496.s004.pdf]
